# Network pharmacology and experimental verification of the mechanism of licochalcone A against *Staphylococcus aureus* pneumonia

**DOI:** 10.3389/fmicb.2024.1369662

**Published:** 2024-05-13

**Authors:** Fengge Shen, Yinghua Zhang, Chunjie Li, Hongyan Yang, Peng Yuan

**Affiliations:** ^1^Xinxiang Key Laboratory of Molecular Neurology, School of Basic Medical Sciences, Xinxiang Medical University, Xinxiang, China; ^2^School of Public Health, Xinxiang Medical University, Xinxiang, China

**Keywords:** licochalcone A, *S. aureus* pneumonia, network pharmacology, anti-infective, NLRP3 inflammasome

## Abstract

*Staphylococcus aureus* strains cause the majority of pneumonia cases and are resistant to various antibiotics. Given this background, it is very important to discover novel host-targeted therapies. Licochalcone A (LAA), a natural plant product, has various biological activities, but its primary targets in *S. aureus* pneumonia remain unclear. Therefore, the purpose of this study was to identify its molecular target against *S. aureus* pneumonia. Network pharmacology analysis, histological assessment, enzyme-linked immunosorbent assays, and Western blotting were used to confirm the pharmacological effects. Network pharmacology revealed 33 potential targets of LAA and *S. aureus* pneumonia. Enrichment analysis revealed that these potential genes were enriched in the Toll-like receptor and NOD-like receptor signaling pathways. The results were further verified by experiments in which LAA alleviated histopathological changes, inflammatory infiltrating cells and inflammatory cytokines (TNF, IL-6, and IL-1β) in the serum and bronchoalveolar lavage fluid *in vivo*. Moreover, LAA treatment effectively reduced the expression levels of NF-κB, p-JNK, p-p38, NLRP3, ASC, caspase 1, IL-1β, and IL-18 in lung tissue. The *in vitro* experimental results were consistent with the *in vivo* results. Thus, our findings demonstrated that LAA exerts anti-infective effects on *S. aureus*-induced lung injury via suppression of the Toll-like receptor and NOD-like receptor signaling pathways, which provides a theoretical basis for understanding the function of LAA against *S. aureus* pneumonia and implies its potential clinical application.

## Introduction

*Staphylococcus aureus (S.aureus)* is a momentous Gram-positive human opportunistic pathogen. Research has shown that it is a serious etiology of community-acquired pneumonia in the last 20 years, with severe community-acquired pneumonia caused by methicillin-resistant *S. aureus* (MRSA) (Self et al., 2016; Ensinck et al., 2021; Sakamoto et al., 2021). MRSA pneumonia patients had greater in-hospital mortality, longer hospital stays and higher healthcare costs. Furthermore, some *S. aureus* strains have even exhibited resistance to in the antibiotic vancomycin, and the number of available vaccine candidates is relatively limited. Thus, novel anti-bacterial agents need to be discovered for alternative prophylaxis and treatments.

*Staphylococcus aureus*-induced pneumonia features a severe inflammatory response in lung tissues. Studies have shown that *S. aureus* can activate the Toll-like receptor 2 (TLR2) signaling pathway, through nuclear factor κ-light-chain-enhancer of activated B cells (NF-κB) and mitogen activated protein kinase (MAPK) to upregulate inflammatory gene expression and release the proinflammatory cytokines interleukin (IL)-6, IL-1β, and tumor necrosis factor (TNF)-α (Calzado et al., 2007; Pidwill et al., 2021). Studies have also shown that *S. aureus* can influence the inflammasome. The NOD-like receptor family pyrin domain containing 3 protein (NLRP3) inflammasome during *S. aureus* lung infection aggravates severe pneumonia pathology (Kebaier et al., 2012). The NLRP3 inflammasome consists of NLRP3, apoptosis-associated speck-like protein containing CARD (ASC), and pro-caspase-1, and has been demonstrated to be activated in *S. aureus.* This activation and splicing of pro-caspase-1 into active caspase-1 (caspase p10 and p20) causes the subsequent proteolytic cleavage of precursor IL-1β and IL-18, which activate IL-1β and active IL-18 (Wang X. et al., 2020). Thus, various strategies such as attenuating exaggerated inflammation by activating the NLRP3 inflammasome, NF-κB or MAPK are under extensive evaluation.

Licochalcone A (LAA; Figure 3A) is a valuable flavonoid of the licorice species *Glycyrrhiza inflata*, and has been demonstrated to have various biological activities, e.g., anti-aging (Wu Y. et al., 2021), anti-obesity (Lee et al., 2018), anti-inflammatory (Phan et al., 2021; Tian et al., 2022), anti-viral (Cao et al., 2021), anti-tumor (Gao et al., 2021), anti-leishmanial (Souza et al., 2020), anti-oxidative (Lv et al., 2018), and hepatoprotective effects (Lv et al., 2018). We also found that LAA has anti-bacterial activity against *S. aureus* biofilms and planktonic cells (Shen et al., 2015). However, the therapeutic effect of LAA against *S. aureus* pneumonia is still unclear.

In this study, a network pharmacology method was used to predict the potential key genes and signaling pathways affected by LAA in the treatment of *S. aureus* pneumonia. Moreover, we established a cell model and a *S. aureus* pneumonia mouse model to further demonstrate the therapeutic mechanism of LAA on *S. aureus* pneumonia.

## Materials and methods

### Ethics statement

All animal experiments were performed according to the “Guide for the Care and Use of Laboratory Animals” and were approved by the Institutional Animal Care and Use Committee of Xinxiang Medical University.

### Materials and reagents

Licochalcone A (>98%) was purchased from Must Bio-Technology Co., Ltd. (Chengdu, China). Seven antibiotic drugs (vancomycin, ciprofloxacin, penicillin G, oxacillin, tetracyclines, levofloxacin, and gentamicin) and hematoxylin and eosin (H&E) kits were purchased from Solarbio Science & Technology Co., Ltd. (Beijing, China). A BCA protein quantitation kit, eECL Western blot kit, RIPA lysis buffer, and LPS were obtained from Beyotime Biotechnology (Haimen, China). The NLRP3 activator ATP (HY-B2176) and TLR2 activator Pam3CSK4 (HY-P1180A) were purchased from MedChemExpress (Shanghai, China). Lactate dehydrogenase assay kit (A020-2-2) was purchased from Nanjing Jiancheng Bioengineering Institute (Nanjing, China). The NLRP3 activator nigericin (Abs819747) was purchased from Absin (Shanghai, China). Phorbol myristate acetate (PMA) was purchased from Sigma-Aldrich. Antibodies against NLRP3 (No. DF7438), IL-1β (No. AF5103), and IL-18 (No. DF6252) were obtained from Affbiotech; antibodies against caspase-1 (14F468) (No. sc-56036) and ASC (F-9) (No. sc-271054) were obtained from Santa Cruz Biotechnology, Inc. (USA), and antibodies against phospho-NF-κB p65 (Ser536, No. AN371), TNF-α (No. AF8208), p-Erk1/2 (No. AF1891), p-JNK (No. AJ516), and p-p38 (No. AM063) were obtained from Beyotime Biotechnology (Haimen, China). Antibodies against GAPDH (No. bs-10900R) were purchased from Beijing Biosynthesis Biotechnology Co., Ltd. (Beijing, China). Mueller-Hinton broth (CA-MHB) was obtained from Hope Bio-Technology Co., Ltd. (Qingdao, China). ELISA kits for the inflammatory cytokines TNF-α (REF 88-7324-22), IL-1β (REF 88-7013-22) and IL-6 (REF 88-7064-22) were purchased from Thermo Fisher Scientific.

### Identification of *S. aureus* pneumonia-related targets of LAA

PharmMapper is an open-source web server that identifies potential drug targets via reversed pharmacophore matching of the query compound against an in-house pharmacophore model database (Wang et al., 2017). The Comparative Toxicogenomics Database (CTD) harmonizes cross-species heterogeneous data for chemical exposures and their biological repercussions by manually curating and interrelating chemical, gene, phenotype, anatomy, disease, taxa, and exposure content from the published literature (Davis et al., 2023). The PharmMapper server^1^ and CTD^2^ were used to collect the LAA-related targets. The *S. aureus* pneumonia-related targets were collected from GeneCards^3^ (Relevance score ≥5) and CTD (see text footnote 2) (Inference score ≥5) with “*Staphylococcus aureus* pneumonia” as the search term. The interactions between the potential targets of LAA and *S. aureus* pneumonia were gathered using the STRING database^4^ with a combined score ≥0.4. The compound-target network was created based on the protein–protein interaction (PPI) network and displayed using Cytoscape-v3.7.2 software. The network characteristics were calculated by the plug-in Network Analyzer of Cytoscape-v3.7.2 software. The degree of freedom was applied as a topological index to indicate the importance of the network node. The larger the value in the network is, the more vital the node.

### Enrichment analysis of LAA and *S. aureus* pneumonia-related target pathways

Gene Ontology (GO) analysis and Kyoto Encyclopedia of Genes and Genomes (KEGG) pathway enrichment analysis were executed using the Database for Annotation, Visualization and Integrated Discovery (DAVID) program.^5^ Specifically, we identified 33 potential targets, selected official_gene_symbol and *Homo sapiens*, and then performed GO analysis and KEGG pathway enrichment analysis. The GO items associated with biological process (BP), cellular component (CC), molecular function (MF), and KEGG signaling pathways were analyzed, and the mapped senior bubbles of the top 15 BP, CC, MF terms and the top 41 KEGG signaling pathways were visualized by Weishengxin^6^ according to the *p* values.

### Molecular docking verification

The chemical structure of LAA was obtained from the Zinc database^7^ and saved in its mol2 format. The three-dimensional structures of intersecting targets were obtained from the RCSB PDB database^8^ in PDB format. The corresponding protein molecular docking was executed using AutoDock Tools 1.5.7. The two-dimensional plan of the docking results was visualized by Discovery Studio 2016 Client.

### Bacterial strains

The standard *S. aureus* strain ATCC 29213 was obtained from the China Medical Culture Collection Center (Beijing, China). *S. aureus* Newman was provided courtesy of Prof. Timothy J. Foster. Five MRSA strains (MRSA 3183, MRSA 2961, MRSA 2047, MRSA 41122, and MRSA 3335) were provided courtesy of Prof. Guo Na of Jilin University, Changchun, China.

### Planktonic antimicrobial susceptibility testing

The minimum inhibitory concentrations (MICs) of LAA and seven antibiotic drugs against the seven *S. aureus* strains were measured by microdilution in cation-adjusted CA-MHB using the Clinical and Laboratory Standards Institute protocol (Clinical and Laboratory Standards Institute [CLSI], 2012a). Oxacillin (2% NaCl) was added to the wells. The MIC was defined as the lowest concentration at which no visible bacterial growth was observed. On the basis of CLSI standards and guidelines, MIC breakpoints were used to define resistant (R), intermediate (I), and susceptible (S) strains (Clinical and Laboratory Standards Institute [CLSI], 2012b). The minimum bactericidal concentrations (MBCs) was defined as the lowest concentration which no microbial growth was detected on agar plates after 24 h at 37°C.

### *S. aureus* challenge

THP-1 cells were differentiated to the macrophage (MΦ) phenotype at 160 ng/mL PMA and then challenged with *S. aureus* (1 × 10^7^ CFU/mL, 2 mL/well) in the absence or presence of LAA for 6 h. The control group did not receive *S. aureus*.

### Mouse model of *S. aureus* pneumonia

Six- to eight week old male BALB/c mice were obtained from Skbex Biotechnology (Henan, China), housed on a 12/12 h light/dark cycle and allowed to eat freely. The *S. aureus* pneumonia model was established by intranasal administration of 1 × 10^9^CFU of *S. aureus* 29213 in 30 μl of sterile isotonic saline, as previously described with modifications (Liu et al., 2017). The mice were randomly divided into the following five groups: control group, *S. aureus* group, and *S. aureus* + LAA (10, 20, and 30 mg/kg) group. The animals were treated intraperitoneally with DMSO or LAA (10, 20, or 30 mg/kg) two times a day. The first LAA injection was 1 h before *S. aureus* infection. Serum, BALF, and lung tissues were obtained 72 h after *S. aureus* infection. The BALF was obtained using a 1 mL syringe with cold PBS. The BALF was loaded onto a slide and stained with Giemsa stain.

### Hematoxylin and eosin staining

Lung, heart, liver, spleen, and kidney samples were obtained and fixed in 4% (w/v) formalin, embedded in paraffin, sliced into 5-mm sections, and stained with hematoxylin and eosin kits according to the manufacturer’s instructions. The specimens were observed and collected with Motic DSAssistant Lite 1.0 software or fluorescence microscopy (Nikon, Japan).

### Lactate dehydrogenase release and cytokine measurements

The serum and BALF from *S. aureus*-induced pneumonia and culture supernatant from THP-1-derived MΦ cells were collected. A lactate dehydrogenase (LDH) cytotoxicity assay kit was used to measure LDH release, and the optical density of the samples was measured with a microplate reader set at 450 nm. The inflammatory cytokines IL-1β, IL-6, and TNF-α were examined using ELISA kits according to the manufacturer’s instructions. Then, the absorbance of each sample was analyzed at 450 nm with a microplate reader (Thermo Scientific, China).

### Western blotting

Total protein from the THP-1 cells and lung tissues was extracted on ice for 10 min with RIPA buffer and then centrifuged at 4°C for 10 min. The protein concentration was calculated using a BCA protein quantification kit. The samples were separated by 8%–12% SDS-PAGE and transferred to a polyvinylidene fluoride membrane (300 mA, 90 min). Then, the membranes were incubated in 5% skim milk for 2 h at room temperature. The membranes were washed with TBST. Subsequently, the membranes were incubated with primary antibodies (1:500) overnight at 4°C. The membranes were incubated with HRP-conjugated secondary antibodies for 1 h at room temperature. The target protein was visualized using ECL Plus detection reagents.

### Statistical analysis

All the statistical analyses in this study were analyzed using SPSS 21 and GraphPad Prism 8 software. The data are presented as the mean ± standard deviation (SD). One-way analysis of variance followed by Dunnett’s *post-hoc* test was used with *p* < 0.05 considered to indicate statistical significance.

## Results

### Activity of LAA against *S. aureus*

The MICs, MBCs, and MIC breakpoints are displayed in Table 1 according to Clinical and Laboratory Standards Institute [CLSI] (2012a,b). *S. aureus* 29213 was sensitive to seven common antibiotics. Five clinical strains of *S. aureus* were sensitive to vancomycin and resistant to penicillin G, oxacillin, ciprofloxacin, levofloxacin, tetracyclines, and gentamicin except for MRSA 41122 (Shen et al., 2020). *S. aureus* Newman strains were sensitive to four common antibiotics (vancomycin, oxacillin, levofloxacin, and gentamicin) and resistant to three common antibiotics (penicillin G, ciprofloxacin, and tetracyclines).

**TABLE 1:** Minimum inhibitory concentrations and minimum bactericidal concentrations of LAA and seven common antibiotics for seven strains of *S. aureus*.

	LAA		PEG		VAN		OXA		CIP		LVX		TET		GEN	
Strain	MIC	MBC	MIC	MBC	MIC	MBC	MIC	MBC	MIC	MBC	MIC	MBC	MIC	MBC	MIC	MBC
*S. aureus* 29213	2	8	0.05 (S)	16	1 (S)	1	<0.125 (S)	2	0.25 (S)	1	<0.25 (S)	2	<0.125 (S)	0.5	1 (S)	1
*S. aureus* Newman	4	8	16 (R)	16	1 (S)	1	<0.0625 (S)	8	4 (R)	8	0.25 (S)	0.25	32 (R)	32	0.25 (S)	0.25
MRSA 3183	4	8	32 (R)	64	l (S)	1	>256 (R)	>256	32 (R)	64	16 (R)	64	128 (R)	128	16 (R)	128
MRSA 2961	4	8	32 (R)	128	l (S)	1	>256 (R)	>256	32 (R)	128	64 (R)	256	16 (R)	32	>256 (R)	>256
MRSA 2047	4	8	16 (R)	32	1 (S)	1	64 (R)	256	16 (R)	64	16 (R)	64	256 (R)	256	256 (R)	>256
MRSA 41122	4	8	32 (R)	32	1 (S)	1	256 (R)	>256	128 (R)	256	64 (R)	128	8 (I)	16	>256 (R)	>256
MRSA 3335	4	8	32 (R)	64	1 (S)	1	>256 (R)	>256	64 (R)	64	64 (R)	128	256 (R)	>256	256 (R)	>256

PEG, penicillin G; VAN, vancomycin; OXA, oxacillin; CIP, ciprofloxacin; LVX, levofloxacin; TET, tetracyclines; GEN, gentamicin; LAA, licochalcone A; S, sensitive; I, intermediate; R, resistant.

The MICs and MBCs of LAA for the target strains were 2–4 and 8 μg/ml, respectively. This suggests that LAA has antimicrobial activity against MRSA indicating that it is a very competitive anti-*S. aureus* drug candidate.

### LAA alleviated pulmonary morphological damage in *S. aureus* pneumonia mice

**FIGURE 1:**
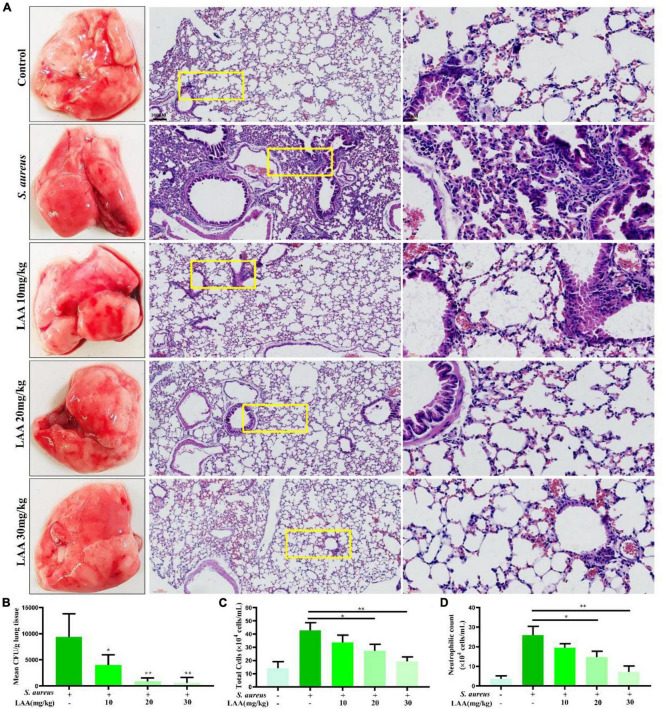
Effects of LAA on *S. aureus*-induced mouse pneumonia. **(A)** The effects of LAA on the pathology and histopathology of mice with *S. aureus pneumonia* (stained with HE, ×10 or ×40). **(B)** CFU counts obtained from lung tissue homogenates. **(C,D)** Inflammatory cellular responses in the lungs of *S. aureus*-infected mice. BALB/c mouse lungs were lavaged. Total inflammatory cells **(C)** and the number of neutrophils **(D)** were enumerated in the BALF. *p < 0.05 and **p < 0.01 between the indicated groups.

To prove the effect of LAA on *S. aureus* pneumonia, we constructed a mouse model of *S. aureus* pneumonia. Histopathological examination showed that the *S. aureus*-infected lungs were slightly or weakly red after LAA treatment. H&E staining of the sliced lung tissues was then performed. The lung tissues of *S. aureus-*infected mice showed thickening of alveolar walls, accumulation of inflammatory cells, edema within alveolar spaces and no intact alveolar structure in some areas. Notably, LAA protected lung tissue with reduced infiltration of inflammatory cells, alveolar wall thickening and edema after infection (Figure 1A). Compared with that in untreated mice, the abundance of *S. aureus* bacteria in the lungs of mice was significantly reduced by LAA (*p* < 0.05; Figure 1B). Moreover, the number of total cells and neutrophils in the BAL fluid increased during *S. aureus* infection. LAA substantially reduced the number of total cells and neutrophils, except at 10 mg/kg (Figures 1C, D). This suggests that LAA alleviated pulmonary morphological damage in *S. aureus* pneumonia mice.

### LAA reduced the expression of IL-6, TNF-α, and IL-1β

**FIGURE 2:**
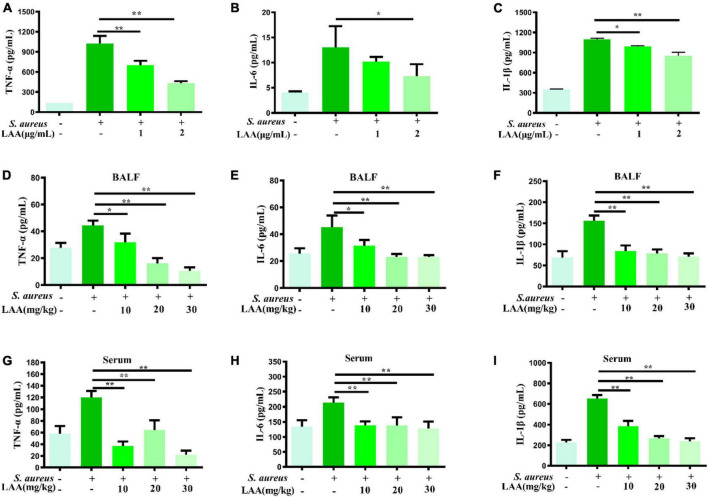
Effects of LAA on proinflammatory cytokines in THP-1 cells, blood and BALF after *S. aureus* infection. THP-1 cells were treated with *S. aureus* in the absence or presence of LAA (1 or 2 μg/mL) for 6 h. The levels of TNF-α **(A)**, IL-6 **(B)**, and IL-1β **(C)** in the culture supernatant of THP-1 cells were tested by ELISA. The levels of TNF-α **(D,G)**, IL-6 **(E,H)**, and IL-1β **(F,I)** in the BALF and serum were detected by ELISA at 72 h after LAA treatment. *n* = 4 in each group. *p < 0.05 and **p < 0.01 between the indicated groups.

To investigate the anti-inflammatory effects of LAA, we detected the levels of IL-6, TNF-α, and IL-1β in culture supernatants from macrophages, serum and BALF by ELISA. As shown in Figures 2A–C, LAA treatment suppressed the *S. aureus*-triggered expression of IL-6, TNF-α, and IL-1β, but not that of IL-6, in THP-1-derived MΦs treated with 1 μg/mL LAA. Similarly, the production of IL-6, TNF-α, and IL-1β was significantly reduced by various concentrations of LAA in the serum and BALF of the mice (*p* < 0.05 and *p* < 0.01; Figures 2D–I). The results suggested that LAA reduced the expression of inflammatory mediators in *S. aureus*-induced macrophages and pneumonia mice.

### Network pharmacology

**FIGURE 3:**
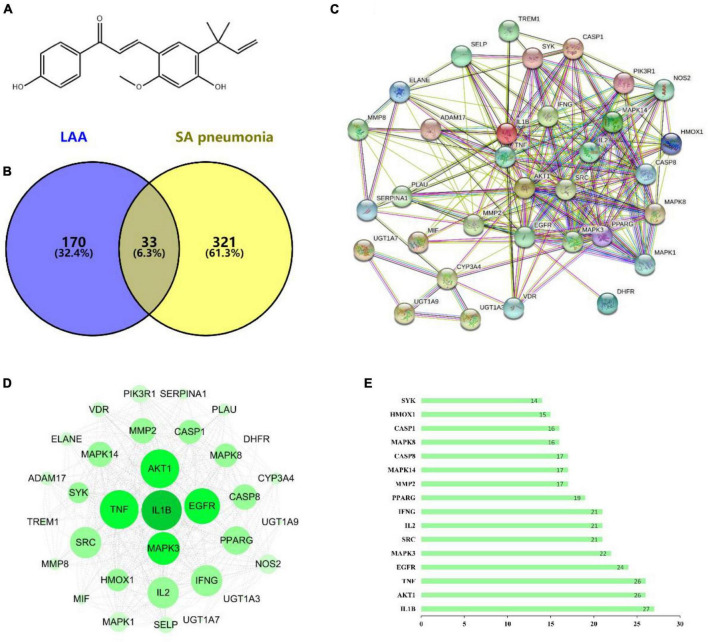
The targets of LAA were collected to explore drug-disease relationships. **(A)** Two-dimensional structure of the LAA structure diagram. **(B)** Venn diagram of LAA and *S. aureus* pneumonia. The blue part represents LAA, and the yellow part represents *S. aureus* pneumonia. **(C)** Disease- drug-active ingredient-key target network. **(D)** PPI network of potential targets of LAA for *S. aureus* pneumonia treatment. **(E)** Bar chart of 16 core genes.

To determine the mechanism underlying the anti-*S. aureus* pneumonia effects of LAA, we used CTD, PharmMapper and GeneCards to identify the *S. aureus* pneumonia-related targets of LAA via text mining. The targets of LAA were identified by PharmMapper and CTD. After deleting duplicates, we ultimately obtained 203 targets (Supplementary Table 1). The *S. aureus* pneumonia-related genes were identified from the GeneCards database and CTD, and 354 *S. aureus* pneumonia-related genes were identified (Supplementary Table 2). The common targets of LAA and *S. aureus* pneumonia-related genes were analyzed using the online software Venny 2.1.0, and Venn diagrams were generated. In this study, 33 genes overlapped as targets of both LAA and *S. aureus* pneumonia (Figure 3B and Supplementary Table 3).

### PPI network analysis

Based on the potential pharmacodynamics of LAA against *S. aureus* pneumonia, the interacting proteins were screened using the STRING online tool. We found 33 nodes with 233 PPI relationships in the network (Figure 3C). The PPI network was established using Cytoscape 3.7.2 software for further visualization and analysis (Figure 3D). A bar chart of the top 16 intersecting targets (including IL1B, AKT1, TNF, EGFR, MAPK3, SRC, IL2, IFNG, PPARG, MMP2, MAPK14, CASP8, MAPK8, CASP1, HMOX1, and SYK) was exhibited according to the degree value, which was greater than or equal to the average score of 13.5 (Figure 3E). All of these results implied that LAA might play an effective role against *S. aureus* pneumonia through these targets.

### GO and KEGG analysis

**FIGURE 4:**
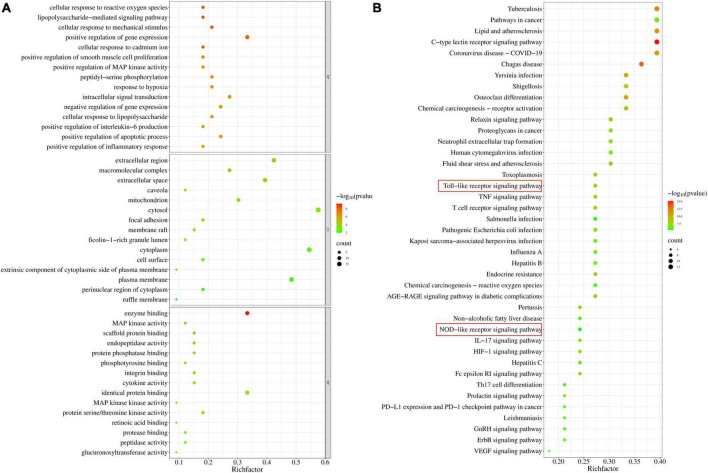
Gene Ontology and KEGG pathway enrichment of candidate targets for LAA in *S. aureus* pneumonia. **(A)** Bubble graph of the GO functional annotation of potential targets of LAA. The top 15 biological process BP, CC, and MF categories were ranked on the basis of –logP values. **(B)** Bubble graph of the top 41 pathways based on KEGG enrichment analysis for potential targets of LAA.

Gene Ontology enrichment analysis was performed with the common potential target genes of LAA and *S. aureus* pneumonia. The top 15 significantly enriched terms in BP, CC, and MF categories are shown in Figure 4A. BP analysis indicated that these targets were interconnected with biological processes, including positive regulation of MAP kinase activity, positive regulation of interleukin-6 production, and positive regulation of the inflammatory response. CC analysis showed that markedly enriched terms were concentrated in the extracellular region, cytosol, and cytoplasm. MF analysis showed that the enriched functional terms included enzyme binding, MAP kinase activity, protein serine/threonine kinase activity, and protease binding.

Furthermore, the 33 targets were enriched in 151 pathways according to KEGG analysis. We observed that these targets participated in pathways including the C-type lectin receptor signaling pathway, the relaxin signaling pathway, the Toll-like receptor signaling pathway, the T cell receptor signaling pathway, the Fc epsilon RI signaling pathway, the TNF signaling pathway, neutrophil extracellular trap formation, and the NOD-like receptor signaling pathway (Figure 4B). In particular, two major pathways, the Toll-like receptor signaling pathway and the NOD-like receptor signaling pathway, are the known therapeutic pathways of *S. aureus* infection. Therefore, the Toll-like receptor and NOD-like receptor signaling pathway-related targets were selected as candidate targets of LAA against *S. aureus* pneumonia for further experimental validation.

### Molecular docking display

**FIGURE 5:**
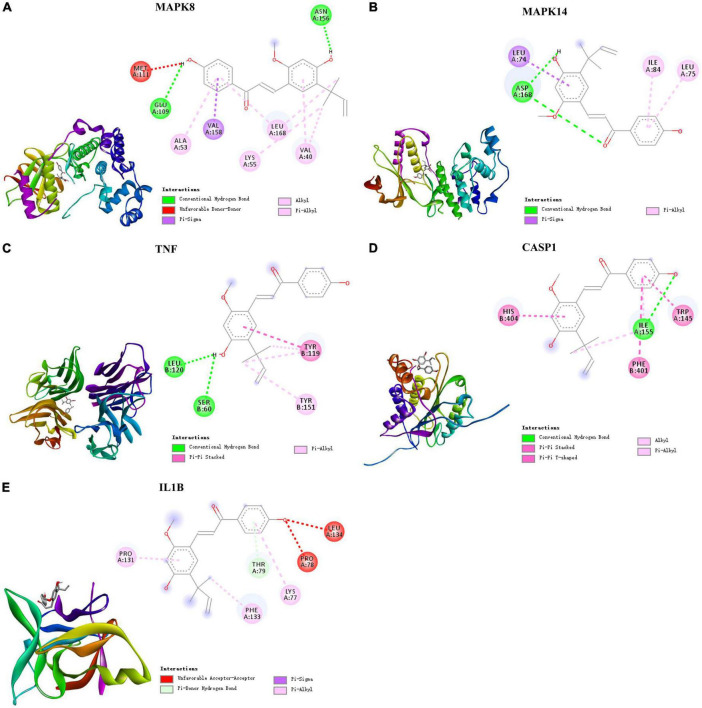
Molecular docking of LAA binding core targets predicted by network pharmacology. LAA binds to proteins **(A)** 3ELJ (MAPK8), **(B)** 7BDO (MAPK14), **(C)** 2AZ5 (TNF), **(D)** 6PZP (CASP1), and **(E)** 6Y8I (IL1B).

Based on these results, the five targets were selected for molecular docking and visualization by AutoDock Vina and Discovery Studio 2016. The details of the docking results are shown in Figure 5 and Table 2.

**TABLE 2:** Different binding energies of LAA with five selected targets.

Rank	Target name	PDB ID	Affinity (kcal/mol)
1	MAPK8	3ELJ	−8.6
2	MAPK14	7BDO	−7.7
3	CASP1	6PZP	−8.1
4	TNF	2AZ5	−8.1
5	IL1B	6Y8I	−6.3

The binding affinities (kcal/mol) of the 5 targets were all < −6 kcal/mol, indicating that LAA had good binding affinity with these receptor proteins. According to the two-dimensional diagram, LAA bound to 3ELJ (MAPK8) through conventional hydrogen bonds with GLU-109 and ASN156. Other forces, including unfavorable donor-donor, pi-sigma, alkyl, and pi-alkyl bonds, were also found. LAA formed 1 conventional hydrogen bond with ASP-168 of 7BDO (MAPK14). In addition, pi-sigma and pi-alkyl bonds also existed. LAA was attracted to 2AZ5 (TNF) by 2 conventional hydrogen bonds with AEU-120 and SER-60, as well as pi-pi stacked and pi-alkyl bonds. When encountering 6PZP (CASP1), LAA formed 1 hydrogen bond with ILE-155, and pi-pi stacked, pi-pi T-shaped, alkyl, and pi-alkyl bonds were also found. When the target was 6Y8I (IL1B), LAA could form unfavorable acceptor-acceptor, pi-donor, pi-sigma, and pi-alkyl hydrogen bonds at the corresponding positions. Combining the results of the free binding energy score and chemical bond distribution showed that LAA might be an inhibitor of these five targets; however, this still needs to be experimentally verified.

### LAA reduced NLRP3 inflammasome activation

**FIGURE 6:**
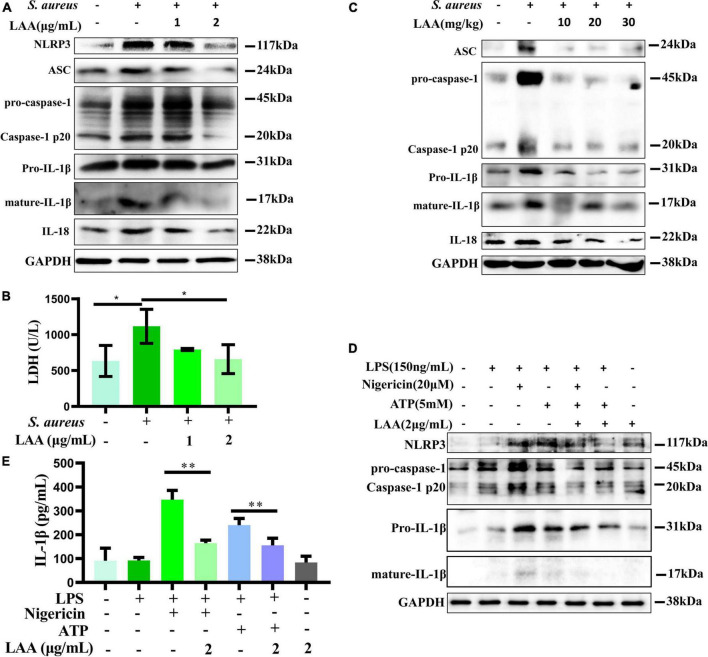
Licochalcone A reduced *S. aureus* induced activation of the NLRP3 inflammasome in THP-1 cells and mouse lung tissue. **(A)** NLRP3, ASC, caspase-1, IL-18, and IL-1β protein expression levels were evaluated by Western blotting in THP-1 cells. **(B)** The concentrations of LDH released by THP-1-derived MΦs were determined using an LDH assay kit to evaluate cell integrity. *n* = 3 in each group. *p < 0.05 between the indicated groups. **(C)** Mice were treated with LAA for 1 h before *S. aureus* infection. The protein expression of ASC, caspase-1, IL-18, and IL-1β in the lung tissue was measured by Western blotting. THP-1-derived MΦs were primed with LPS and then stimulated with LAA for 6 h with or without the NLRP3 inflammasome activator nigericin or ATP for 0.5 h before the end of the experiment. **(D)** NLRP3, caspase-1 and IL-1β protein expression levels were evaluated by Western blotting. **(E)** The levels of IL-1β were tested by ELISA. *n* = 4 in each group. **p < 0.01 between the indicated groups.

To survey the inhibitory effect of LAA on activated MΦs, THP-1-derived MΦs were used for *in vitro* experiments. *In vitro*, we investigated the activation of the NLRP3 inflammasome (NLRP3, ASC, caspase-1, IL-1β, and IL-18) in MΦs after treatment with *S. aureus* and LAA. As shown in Figure 6A, the levels of NLRP3, ASC, pro-caspase-1, caspase-1 p20, pro-IL-1β, mature-IL-1β, and IL-18 were decreased by LAA in *S. aureus-*treated THP-1-derived MΦ compared to *S. aureus* alone. The concentrations of LDH released from THP-1-derived MΦs were determined using an LDH assay kit to evaluate cell integrity. As shown in Figure 6B, *S. aureus* increased LDH release compared to control group (*p* < 0.05), and 2 μg/mL LAA co-treatment decreased LDH release in *S. aureus-*treated THP-1-derived MΦs (*p* < 0.05); however, the effect of 1 μg/mL LAA was not obvious. These findings suggested that LAA reduced *S. aureus*-induced NLRP3 inflammasome activation *in vitro*. Moreover, LAA treatment reduced ASC, pro-caspase-1, caspase-1 p20, pro-IL-1β, mature-IL-1β, and IL-18 activation in the lungs of pneumonia mice in comparison with *S. aureus*-treated mice (Figure 6C). These results suggest that LAA reduced the activation of the NLRP3 inflammasome induced by *S. aureus* in macrophages and in mice with pneumonia.

To demonstrate that the anti-inflammatory effect of LAA is not solely dependent on its antibacterial effect, we further examined the effect of LAA on ATP and nigericin-induced NLRP3 inflammasome *in vitro*. As shown in Figure 6D, compared with those in the control group, the levels of NLRP3, pro-caspase-1, caspase-1 p20, pro-IL-1β, and mature-IL-1β in the THP-1-derived MΦ were increased by ATP or nigericin, and the levels of these proteins were decreased with LAA treatment compared to ATP or nigericin in THP-1-derived MΦ. Meanwhile, Figure 6E indicated that LAA treatment suppressed ATP- or nigericin-induced expression of IL-1β in the culture supernatant of THP-1-derived MΦs (*p* < 0.01). LAA treatment alone was not significant for these indicators (Figures 6D, E). This finding suggested that LAA can inhibit NLRP3 inflammasome activation independently of antibacterial activity.

### LAA inhibited TLR signaling activation

**FIGURE 7:**
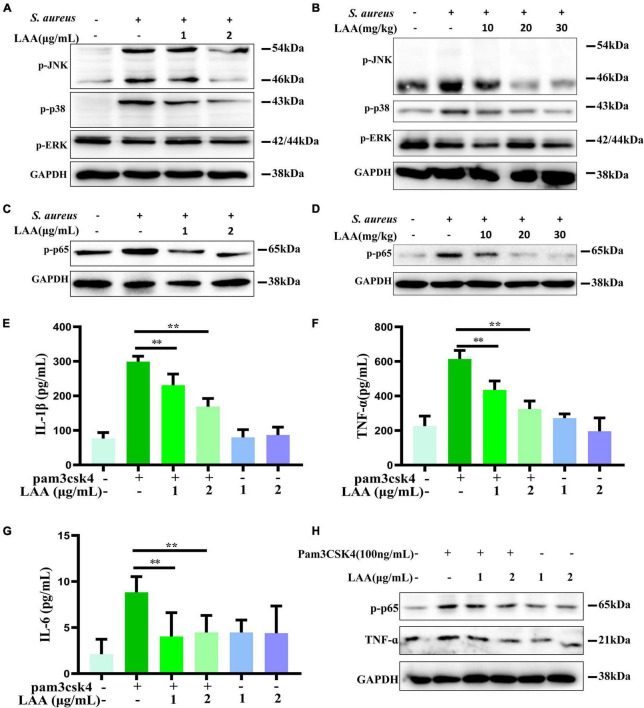
The effect of LAA on MAPK and NF-κB pathway activation. **(A)** The levels of p-JNK, p-p38, and p-ERK in THP-1 cells were evaluated by Western blotting. **(B)** The levels of p-JNK, p-p38, and p-ERK in the lung tissue. **(C)** p-p65 protein expression in THP-1 cells. **(D)** p-p65 protein expression in lung tissues. **(E–G)** THP-1-derived MΦs were treated with the TLR2 activator Pam3CSK4 in the absence or presence of LAA for 6 h. The levels of IL-1β **(E)**, TNF-α **(F)**, and IL-6 **(G)** were tested by ELISA. *n* = 4 in each group. **p < 0.01 between the indicated groups. **(H)** The expression levels of p-p65 and TNF-α were evaluated by Western blotting.

*Staphylococcus aureus* can activate the TLR2 signaling pathway through NF-κB and MAPK to upregulate inflammatory gene expression. Next, we assessed the *in vitro* anti-inflammatory activities of LAA in THP-1-derived MΦs that were activated by *S. aureus*. As shown in Figure 7A, the p-JNK and p-p38 levels, but not the p-ERK level, in THP-1-derived macrophages were increased by *S. aureus*, whereas LAA inhibited the *S. aureus*-induced phosphorylation of JNK and p38 MAPK in THP-1-derived MΦ (Figure 7A). As shown in Figure 7B, *S. aureus* treatment increased p-JNK and p-p38 levels but not ERK levels in the lungs of the mice. In contrast, LAA treatment markedly reduced JNK and p38 phosphorylation, but not ERK phosphorylation (Figure 7B). These results showed that LAA reduced JNK and p38MAPK signaling activation in macrophages and in *S. aureus*-induced pneumonia mice.

We found that the phosphorylation of NF-κB was induced by *S. aureus*, suggesting that the NF-κB signaling axis was activated (Figure 7C). Interestingly, LAA inhibited the *S. aureus*-induced phosphorylation of NF-κB in *S. aureus*-induced THP-1-derived MΦs (Figure 7C). Moreover, LAA treatment inhibited the phosphorylation of NF-κB in the lungs of pneumonia mice compared with that in the lungs of *S. aureus*-treated mice (Figure 7D). These results showed that LAA had an anti-inflammatory effect by reducing NF-κB in *S. aureus*-induced macrophages and in mice with pneumonia.

To further prove the inhibitory effect of LAA on the TLR signaling pathway, the TLR2 activator Pam3CSK4 was used for *in vitro* experiments. As shown in Figures 7E–G, the levels of IL-1β, TNF-α, and IL-6 in the culture supernatant of THP-1-derived MΦs were greater in the Pam3CSK4 group than in the control group, and the levels of these proinflammatory cytokines were lower in the LAA group than in the Pam3CSK4 group (*p* < 0.01). Moreover, as shown in Figure 7H, LAA treatment suppressed the Pam3CSK4-induced increase in p-p65 and TNF-α levels in the THP-1-derived MΦs. LAA treatment alone was not significant for these indicators (Figures 7E–H). This finding suggested that LAA has an inhibitory effect on the *S. aureus*-induced TLR signaling pathway *in vitro*.

### Effects of LAA on organ damage

**FIGURE 8:**
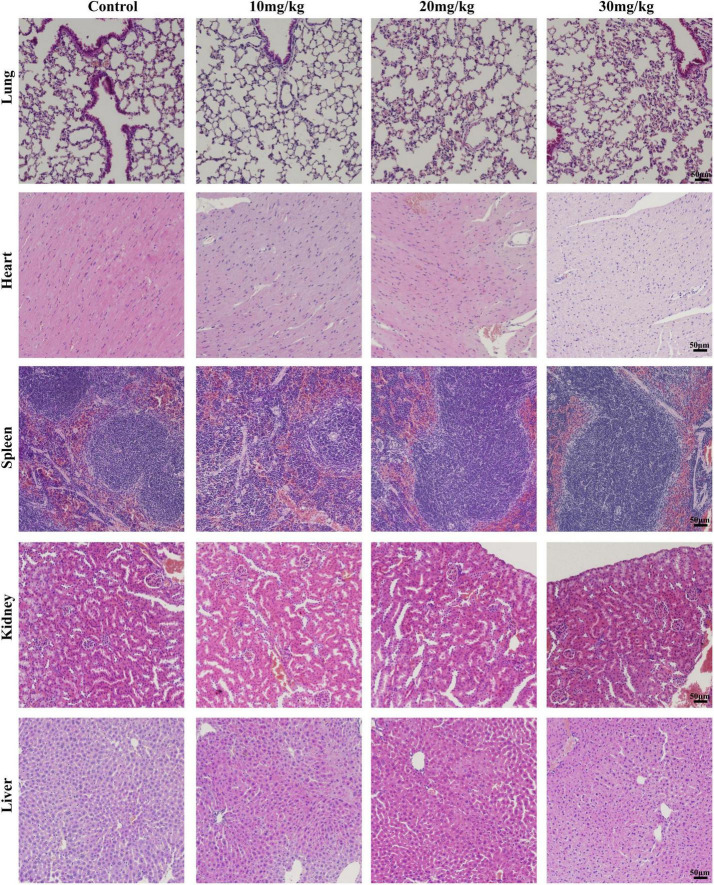
Hematoxylin and eosin staining images of organs including the heart, liver, spleen, lung, and kidney (×20). The mice were intraperitoneally injected with either 0.9% saline or LAA (at dosages of 10, 20, or 30 mg/kg) twice daily, consistent with the drug administration protocol used in the infection model. Seventy-two hours after administration, the heart, liver, spleen, lung, and kidney tissues of the mice were collected, fixed, sliced, and stained.

To investigate the organ damage of the LAA during the time of the subject, the histology of vital organs (heart, liver, spleen, lung, and kidney) was examined by H&E. No obvious signs of damage, such as inflammation, necrosis, pyknosis, polymorphonuclear infiltration, or interstitial hemorrhage, were observed in any of the examined organs, including the heart, liver, spleen, lung, and kidney, after LAA treatment (Figure 8). Our results suggest that LAA has no significant organ damage to mice within 3 days.

## Discussion

Natural compounds have the advantages of biological activity and diversity and have been historically recognized as major sources of traditional medicines and new drug discovery (Boufridi and Quinn, 2018; Ekiert and Szopa, 2020). Natural products have been used since antiquity as traditional medicines and in the modern era. Increasing evidence indicates that natural products play a crucial role in the treatment of *S. aureus* pneumonia (Shaukat et al., 2019; Yao and Sun, 2019; Wu Y. X. et al., 2021). Notably, these drugs displayed multitargeting properties and lower systemic toxicity.

Licochalcone A is isolated from *Glycyrrhiza inflata*, which is widely used clinically in traditional Chinese medicine. A previous study demonstrated that 20–80 mg/kg LAA had obvious anti-inflammatory effects on LPS-induced acute lung injury (Chu et al., 2013). Our previous studies and other previous studies have found that LAA inhibits *S. aureus* activity and the secretion of enterotoxins A and B by *S. aureus* (Qiu et al., 2010; Shen et al., 2015). However, it is unclear whether LAA plays a vital role in the treatment of *S. aureus* infection, especially *S. aureus* pneumonia; this leads to the following question, what is the underlying mechanism of by which LAA affects *S. aureus* pneumonia?

Our research (Table 1) and others’ research revealed that *S. aureus* has developed resistance to many commonly used antibiotics (Parker et al., 2016; Shen et al., 2020), which is an urgent problem to be solved in clinical practice. Notably, LAA inhibited *S. aureus* activity and is an extremely valuable drug candidate for the treatment of *S. aureus* infections. Further analysis suggested that LAA has a protective effect against *S. aureus* pneumonia in mice by changing the histopathology and number of *S. aureus* bacteria in the lungs, and the number of total cells and neutrophils in the BAL fluid (Figure 1).

*Staphylococcus aureus* infection induces severe inflammation, which leads to significant upregulation of TNF-α, IL-1β, and IL-6 in both lung tissues and cells (Yao and Sun, 2019). Thus, we tested the levels of IL-6, TNF-α, and IL-1β in culture supernatants from macrophages, serum and BALF, and the results showed that LAA reduced the levels of IL-6, TNF-α, and IL-1β in culture supernatants from *S. aureus*-induced macrophages and serum and BALF of pneumonia mice (Figure 2). Our results showed that LAA might have therapeutic potential by modulating inflammatory responses in *S. aureus* infection.

Next, the possible mechanism of LAA in *S. aureus* pneumonia was predicted by network pharmacology. Thirty-three shared targets of LAA and *S. aureus* pneumonia-related genes overlapped (Figure 3C). These targets were enriched in terms and pathways identified using GO and KEGG analyses. The Toll-like receptor and NOD-like receptor signaling pathways were identified found (Figure 4B). Therefore, the Toll-like receptor and NOD-like receptor signaling pathway-related targets were selected as candidate targets of LAA against *S. aureus* pneumonia for further molecular docking and experimental validation.

Two pattern recognition receptors, TLRs and NLRs, are mainly responsible for *S. aureus* infection (Philpott et al., 2014). NLRs are capable of forming inflammasomes in response to *S. aureus*, and their composition includes *S. aureus* extracellular vesicles, α-toxin, Panton-Valentine leucocidin, etc. (Holzinger et al., 2012; Wang X. et al., 2020; Wang et al., 2022). NLRP3, which plays a vital role in the host immune response to *S. aureus*, is one of the most characterized members of the NLR family (Duewell et al., 2010; Wang et al., 2022).

There is cumulative evidence indicating that the NLRP3 inflammasome is an attractive therapeutic target in inflammatory diseases (Wang Z. et al., 2020; Seok et al., 2021; Vong et al., 2021). In recent years, the role of the NLRP3 inflammasome in *S. aureus* pneumonia has been studied (Wu and Huang, 2017; Duan et al., 2021). It is widely known that activation of the NLRP3 inflammasome leads to caspase 1-mediated proteolytic activation of IL-1β and results in inflammatory responses (Mangan et al., 2018). In this study, LAA treatment reduced the protein expression of the NLRP3 inflammasome in the lung tissue of *S. aureus* pneumonia mice.

TLRs are essential for defense against *S. aureus* infection. TLR activation by *S. aureus* promotes the recruitment of adaptor proteins to activate NF-κB and MAPKs. NF-κB plays a crucial role in the amplification of inflammation (Liu et al., 2020). Activated NF-κB and MAPKs stimulate *S. aureus-*infected cells to produce numerous inflammatory factors, such as TNF-α, IL-1β, and IL-6 (Shamsuddin and Kumar, 2011; Wu Y. X. et al., 2021).

Previous research has suggested that *S. aureus* infection can upregulate the phosphorylation of p65 proteins, and activate the NF-κB signaling pathway in lung tissues (Wu Y. X. et al., 2021). Our results showed that the level of p-p65 protein in THP-1-derived MΦs and lung tissues was upregulated significantly after *S. aureus* infection, and LAA treatment markedly downregulated the phosphorylation of NF-κB in the lungs of pneumonia mice and in THP-1-derived MΦs.

The MAPK signaling pathway plays crucial roles in the *S. aureus*-induced TLR2-mediated inflammatory response (Jiang et al., 2017). In this study, LAA attenuated the levels of p-JNK and p-p38MAPK in *S. aureus*-infected lungs and THP-1-derived MΦs (Figures 7A, B). However, the phosphorylation level of ERK was not changed by *S. aureus* and/or LAA cotreatment in the lung or in THP-1-derived macrophages. Similar evidence has been found in previous studies (Deramaudt et al., 2020).

In this study, the numbers of *S. aureus* (CFU counts) were strongly reduced by LAA in the lung tissue (Figure 1B). Meanwhile, *in vitro* experiments also showed that LAA significantly enhanced the bactericidal effect of phagocytes on *S. aureus* (*p* < 0.01; Supplementary Figure 1). Obviously, the reduction of bacteria caused by LAA could alleviate inflammation. The crucial question was whether LAA had an anti-inflammatory effect that was independent of its anti-bacterial effect. To further prove the inhibitory effect of LAA on NLRP3 inflammasome and TLR signaling pathways, the NLRP3 inflammasome activator ATP and nigericin and the TLR2 activator Pam3CSK4 were used for *in vitro* experiments. The levels of NLRP3, pro-caspase-1, caspase-1 p20, pro-IL-1β, and mature-IL-1β decreased with LAA treatment in ATP- or nigericin-activated MΦ, and the levels of p-p65, IL-1β, TNF-α, and IL-6 decreased with LAA treatment in Pam3CSK4-activated MΦ (Figures 6, 7). In summary, LAA inhibits *S. aureus* infection by reducing the numbers of *S. aureus* and inhibiting the NLRP3 inflammasome and TLR signaling.

The safety of drugs is the focus of researchers. According to research findings, LAA reduced pre-neoplastic lesions induced by 1,2-dimethylhydrazine in the rat colon at 3.12–50 mg/kg b.w. for 15 days, while biochemical markers and body weight indicated no apparent toxicity (de Freitas et al., 2020). LAA (100 mg/kg) has protective effects against acetaminophen-induced hepatotoxicity, while histological assessment of the mice treated with LAA alone indicated no apparent toxicity (Lv et al., 2018). We conducted a 3-day animal toxicity test for LAA, and found that vital organs including the heart, liver, spleen, lung, and kidney had no obvious signs of damage, such as inflammation, necrosis, pyknosis, polymorphonuclear infiltration, or interstitial hemorrhage in LAA treated mice compared to normal mice (Figure 8). Our results suggest that LAA has no significant damage to mice within 3 days. Based on the literature and the results of LAA only treatment experiments, these findings suggest that LAA at the subject concentration is a safe drug for treating *S. aureus* pneumonia. Currently, clinical research on LAA has focused primarily on its application in the treatment of skin diseases, and further clinical investigations are needed to explore its potential for the systemic treatment of diseases such as *S. aureus* pneumonia.

## Conclusion

In summary, network pharmacology and experimental results showed that LAA protected against *S. aureus* pneumonia in mice by inhibiting NF-κB, JNK, p38MAPK, and NLRP3-mediated inflammation. The results showed that LAA is a potential agent for the treatment of *S. aureus* pneumonia.

## Data availability statement

The original contributions presented in this study are included in this article/Supplementary material, further inquiries can be directed to the corresponding author.

## Ethics statement

Ethical approval was not required for the study involving humans in accordance with the local legislation and institutional requirements. Written informed consent to participate in this study was not required from the participants or the participants’ legal guardians/next of kin in accordance with the national legislation and the institutional requirements. The animal study was approved by the Institutional Animal Care and Use Committee of Xinxiang Medical University. The study was conducted in accordance with the local legislation and institutional requirements.

## Author contributions

FS: Conceptualization, Investigation, Methodology, Writing – original draft. YZ: Data curation, Methodology, Writing – review & editing. CL: Data curation, Methodology, Writing – review & editing. HY: Investigation, Writing – review & editing. PY: Conceptualization, Writing – review & editing.

## Footnotes

1
http://www.lilab-ecust.cn/pharmmapper/

2
http://ctdbase.org/

3
https://www.genecards.org/

4
https://string-db.org/

5
https://david.ncifcrf.gov/

6
http://www.bioinformatics.com.cn/

7
https://zinc.docking.org/

8
http://www.rcsb.org/

## References

[B1] Boufridi A. Quinn R. J. (2018). Harnessing the properties of natural products. *Annu. Rev. Pharmacol. Toxicol.* 58 451–470. 10.1146/annurev-pharmtox-010716-105029 28968192

[B2] Calzado M. A. Bacher S. Schmitz M. L. (2007). NF-kappaB inhibitors for the treatment of inflammatory diseases and cancer. *Curr. Med. Chem.* 14 367–376. 10.2174/092986707779941113 17305539

[B3] Cao Y. Lei E. Wang X. Qi X. Li L. Ren J. (2021). Licochalcone A inhibits enterovirus A71 replication *in vitro* and *in vivo*. *Antiviral. Res.* 195:105091. 10.1016/j.antiviral.2021.105091 34044060

[B4] Chu X. Jiang L. Wei M. Yang X. Guan M. Xie X. (2013). Attenuation of allergic airway inflammation in a murine model of asthma by Licochalcone A. *Immunopharmacol. Immunotoxicol.* 35 653–661. 10.3109/08923973.2013.834929 24028304

[B5] Clinical and Laboratory Standards Institute [CLSI] (2012a). *Methods for dilution antimicrobial susceptibility tests for bacteria that grow aerobically, approved standard*, Document M07-A9. Wayne, PA: CLSI.

[B6] Clinical and Laboratory Standards Institute [CLSI] (2012b). *Clinical and Laboratory Standards Institute. Performance standards for antimicrobial susceptibility testing. Twenty-second informational supplement.* Document M100-S22. Wayne, PA: CLSI.

[B7] Davis A. P. Wiegers T. C. Johnson R. J. Sciaky D. Wiegers J. Mattingly C. J. (2023). Comparative toxicogenomics database (CTD): Update 2023. *Nucleic Acids Res.* 51 D1257–D1262. 10.1093/nar/gkac833 36169237 PMC9825590

[B8] de Freitas K. S. Squarisi I. S. Acésio N. O. Nicolella H. D. Ozelin S. D. Reis Santos de Melo M. (2020). Licochalcone A, a licorice flavonoid: Antioxidant, cytotoxic, genotoxic, and chemopreventive potential. *J. Toxicol. Environ. Health A* 83 673–686. 10.1080/15287394.2020.1813228 32886024

[B9] Deramaudt T. B. Ali M. Vinit S. Bonay M. (2020). Sulforaphane reduces intracellular survival of *Staphylococcus aureus* in macrophages through inhibition of JNK and p38 MAPK-induced inflammation. *Int. J. Mol. Med.* 45 1927–1941. 10.3892/ijmm.2020.4563 32323751 PMC7169961

[B10] Duan W. Qin F. Wu D. Dai Y. (2021). Diphenyl pyrimidine exhibits protective effect on *Staphylococcus aureus* pneumonia in rat model by targeting NLRP3 expression. *Microb. Pathog.* 161(Pt A):105168. 10.1016/j.micpath.2021.105168 34478857

[B11] Duewell P. Kono H. Rayner K. J. Sirois C. M. Vladimer G. Bauernfeind F. G. (2010). NLRP3 inflammasomes are required for atherogenesis and activated by cholesterol crystals. *Nature* 464 1357–1361. 10.1038/nature08938 20428172 PMC2946640

[B12] Ekiert H. M. Szopa A. (2020). Biological activities of natural products. *Molecules* 25:5769. 10.3390/molecules25235769 33297511 PMC7730830

[B13] Ensinck G. Lazarte G. Ernst A. Romagnoli A. López Papucci S. Aletti A. (2021). Community-acquired methicillin-resistant *Staphylococcus aureus* pneumonia in a children’s hospital. Our ten-year experience. *Arch. Argent. Pediatr.* 119 11–17. 10.5546/aap.2021.eng.11 33458975

[B14] Gao F. Li M. Yu X. Liu W. Zhou L. Li W. (2021). Licochalcone A inhibits EGFR signalling and translationally suppresses survivin expression in human cancer cells. *J. Cell Mol. Med.* 25 813–826. 10.1111/jcmm.16135 33247550 PMC7812290

[B15] Holzinger D. Gieldon L. Mysore V. Nippe N. Taxman D. J. Duncan J. A. (2012). *Staphylococcus aureus* panton-valentine leukocidin induces an inflammatory response in human phagocytes via the NLRP3 inflammasome. *J. Leukoc. Biol.* 92 1069–1081. 10.1189/jlb.0112014 22892107 PMC3476237

[B16] Jiang K. F. Zhao G. Deng G. Z. Wu H. C. Yin N. N. Chen X. Y. (2017). Polydatin ameliorates *Staphylococcus aureus*-induced mastitis in mice via inhibiting TLR2-mediated activation of the p38 MAPK/NF-κB pathway. *Acta Pharmacol. Sin.* 38 211–222. 10.1038/aps.2016.123 27890916 PMC5309755

[B17] Kebaier C. Chamberland R. R. Allen I. C. Gao X. Broglie P. M. Hall J. D. (2012). *Staphylococcus aureus* α-hemolysin mediates virulence in a murine model of severe pneumonia through activation of the NLRP3 inflammasome. *J. Infect. Dis.* 205 807–817. 10.1093/infdis/jir846 22279123 PMC3274379

[B18] Lee H. E. Yang G. Han S. H. Lee J. H. An T. J. Jang J. K. (2018). Anti-obesity potential of Glycyrrhiza uralensis and licochalcone A through induction of adipocyte browning. *Biochem. Biophys. Res. Commun.* 503 2117–2123. 10.1016/j.bbrc.2018.07.168 30093114

[B19] Liu K. Ding T. Fang L. Cui L. Li J. Meng X. (2020). Organic selenium ameliorates *Staphylococcus aureus*-induced mastitis in rats by inhibiting the activation of NF-κB and MAPK signaling pathways. *Front. Vet. Sci.* 7:443. 10.3389/fvets.2020.00443 32851026 PMC7406644

[B20] Liu S. Liu B. Luo Z. Q. Qiu J. Zhou X. Li G. (2017). The combination of osthole with baicalin protects mice from *Staphylococcus aureus* pneumonia. *World J. Microbiol. Biotechnol.* 33:11. 10.1007/s11274-016-2162-9 27878749

[B21] Lv H. Xiao Q. Zhou J. Feng H. Liu G. Ci X. (2018). Licochalcone A upregulates Nrf2 antioxidant pathway and thereby alleviates acetaminophen-induced hepatotoxicity. *Front. Pharmacol.* 9:147. 10.3389/fphar.2018.00147 29628888 PMC5876234

[B22] Mangan M. S. J. Olhava E. J. Roush W. R. Seidel H. M. Glick G. D. Latz E. (2018). Targeting the NLRP3 inflammasome in inflammatory diseases. *Nat. Rev. Drug Discov.* 17:688. 10.1038/nrd.2018.149 30116046

[B23] Parker D. Ahn D. Cohen T. Prince A. (2016). Innate immune signaling activated by MDR bacteria in the airway. *Physiol. Rev.* 96 19–53. 10.1152/physrev.00009.2015 26582515 PMC4698397

[B24] Phan H. T. L. Kim H. J. Jo S. Kim W. K. Namkung W. Nam J. H. (2021). Anti-inflammatory effect of licochalcone A via regulation of ORAI1 and K+ channels in T-lymphocytes. *Int. J. Mol. Sci.* 22:10847. 10.3390/ijms221910847 34639190 PMC8509259

[B25] Philpott D. J. Sorbara M. T. Robertson S. J. Croitoru K. Girardin S. E. (2014). NOD proteins: Regulators of inflammation in health and disease. *Nat. Rev. Immunol.* 14 9–23. 10.1038/nri3565 24336102

[B26] Pidwill G. R. Gibson J. F. Cole J. Renshaw S. A. Foster S. J. (2021). The role of macrophages in *Staphylococcus aureus* infection. *Front. Immunol.* 11:620339. 10.3389/fimmu.2020.620339 33542723 PMC7850989

[B27] Qiu J. Feng H. Xiang H. Wang D. Xia L. Jiang Y. (2010). Influence of subinhibitory concentrations of licochalcone A on the secretion of enterotoxins A and B by *Staphylococcus aureus*. *FEMS Microbiol. Lett.* 307 135–141. 10.1111/j.1574-6968.2010.01973.x 20412304

[B28] Sakamoto Y. Yamauchi Y. Jo T. Michihata N. Hasegawa W. Takeshima H. (2021). In-hospital mortality associated with community-acquired pneumonia due to methicillin-resistant *Staphylococcus aureus*: A matched-pair cohort study. *BMC Pulm. Med.* 21:345. 10.1186/s12890-021-01713-1 34732194 PMC8564271

[B29] Self W. H. Wunderink R. G. Williams D. J. Zhu Y. Anderson E. J. Balk R. A. (2016). *Staphylococcus aureus* community-acquired pneumonia: Prevalence, clinical characteristics, and outcomes. *Clin. Infect. Dis.* 63 300–309. 10.1093/cid/ciw300 27161775 PMC4946021

[B30] Seok J. K. Kang H. C. Cho Y. Y. Lee H. S. Lee J. Y. (2021). Therapeutic regulation of the NLRP3 inflammasome in chronic inflammatory diseases. *Arch. Pharm. Res.* 44 16–35. 10.1007/s12272-021-01307-9 33534121 PMC7884371

[B31] Shamsuddin N. Kumar A. (2011). TLR2 mediates the innate response of retinal Muller glia to *Staphylococcus aureus*. *J. Immunol.* 186 7089–7097. 10.4049/jimmunol.1100565 21602496 PMC3110513

[B32] Shaukat A. Guo Y. F. Jiang K. Zhao G. Wu H. Zhang T. (2019). Ginsenoside Rb1 ameliorates *Staphylococcus aureus*-induced acute lung injury through attenuating NF-κB and MAPK activation. *Microb. Pathog.* 132 302–312. 10.1016/j.micpath.2019.05.003 31059756

[B33] Shen F. Ge C. Yuan P. (2020). Metabolomics study reveals inhibition and metabolic dysregulation in *Staphylococcus aureus* planktonic cells and biofilms induced by carnosol. *Front. Microbiol.* 11:538572. 10.3389/fmicb.2020.538572 33072009 PMC7530940

[B34] Shen F. Tang X. Wang Y. Yang Z. Shi X. Wang C. (2015). Phenotype and expression profile analysis of *Staphylococcus aureus* biofilms and planktonic cells in response to licochalcone A. *Appl. Microbiol. Biotechnol.* 99 359–373. 10.1007/s00253-014-6076-x 25256617

[B35] Souza J. M. de Carvalho ÉA. A. Candido A. C. B. B. de Mendonça R. P. Fernanda da Silva M. Parreira R. L. T. (2020). Licochalcone a exhibits leishmanicidal activity *in vitro* and in experimental model of leishmania (Leishmania) infantum. *Front. Vet. Sci.* 7:527. 10.3389/fvets.2020.00527 33363224 PMC7758436

[B36] Tian M. Li N. Liu R. Li K. Du J. Zou D. (2022). The protective effect of licochalcone A against inflammation injury of primary dairy cow claw dermal cells induced by lipopolysaccharide. *Sci. Rep.* 12:1593. 10.1038/s41598-022-05653-6 35102233 PMC8803976

[B37] Vong C. T. Tseng H. H. L. Yao P. Yu H. Wang S. Zhong Z. (2021). Specific NLRP3 inflammasome inhibitors: Promising therapeutic agents for inflammatory diseases. *Drug Discov. Today* 26 1394–1408. 10.1016/j.drudis.2021.02.018 33636340

[B38] Wang X. Eagen W. J. Lee J. C. (2020). Orchestration of human macrophage NLRP3 inflammasome activation by *Staphylococcus aureus* extracellular vesicles. *Proc. Natl. Acad. Sci. U.S.A.* 117 3174–3184. 10.1073/pnas.1915829117 31988111 PMC7022218

[B39] Wang X. Liu M. Geng N. Du Y. Li Z. Gao X. (2022). *Staphylococcus aureus* mediates pyroptosis in bovine mammary epithelial cell via activation of NLRP3 inflammasome. *Vet. Res.* 53:10. 10.1186/s13567-022-01027-y 35123552 PMC8817610

[B40] Wang X. Shen Y. Wang S. Li S. Zhang W. Liu X. (2017). PharmMapper 2017 update: A web server for potential drug target identification with a comprehensive target pharmacophore database. *Nucleic Acids Res.* 45 W356–W360. 10.1093/nar/gkx374 28472422 PMC5793840

[B41] Wang Z. Zhang S. Xiao Y. Zhang W. Wu S. Qin T. (2020). NLRP3 inflammasome and inflammatory diseases. *Oxid. Med. Cell Longev.* 2020:4063562. 10.1155/2020/4063562 32148650 PMC7049400

[B42] Wu S. Huang J. (2017). Resveratrol alleviates *Staphylococcus aureus* pneumonia by inhibition of the NLRP3 inflammasome. *Exp. Ther. Med.* 14 6099–6104. 10.3892/etm.2017.5337 29285164 PMC5740584

[B43] Wu Y. X. Jiang F. J. Liu G. Wang Y. Y. Gao Z. Q. Jin S. H. (2021). Dehydrocostus lactone attenuates methicillin-resistant *Staphylococcus aureus*-induced inflammation and acute lung injury via modulating macrophage polarization. *Int. J. Mol. Sci.* 22:9754. 10.3390/ijms22189754 34575918 PMC8472345

[B44] Wu Y. Wang H. Zhu J. Shen H. Liu H. (2021). Licochalcone A activation of glycolysis pathway has an anti-aging effect on human adipose stem cells. *Aging (Albany NY)* 13 25180–25194. 10.18632/aging.203734 34862330 PMC8714166

[B45] Yao L. Sun T. (2019). Glycyrrhizin administration ameliorates *Streptococcus aureus*-induced acute lung injury. *Int. Immunopharmacol.* 70 504–511. 10.1016/j.intimp.2019.02.046 30884430

