# Supplementary material for: Network pharmacology and experimental verification of the mechanism of licochalcone A against Staphylococcus aureus pneumonia
Source: Front Microbiol. 2024 May 13;15:1369662. doi: 10.3389/fmicb.2024.1369662 (PMC11128579; doi:10.3389/fmicb.2024.1369662)
Supplement: Supplementary file 1 [file Data_Sheet_1.docx]

Supplementary Material

Network Pharmacology and Experimental Verification of the Mechanism of Licochalcone A against Staphylococcus aureus pneumonia

Fengge Shen^1^, Yinghua Zhang^1^, Chunjie Li^1^, Hongyan Yang^1^ and Peng Yuan^2^*

^1^Xinxiang Key Laboratory of Molecular Neurology, School of Basic Medical Sciences, Xinxiang Medical University, Xinxiang 453003, China

^2^School of Public Health, Xinxiang Medical University, Xinxiang 453003, China

*Corresponding author: Peng Yuan, yuanpeng0226@126.com

Table 1S. LAA associated genes.


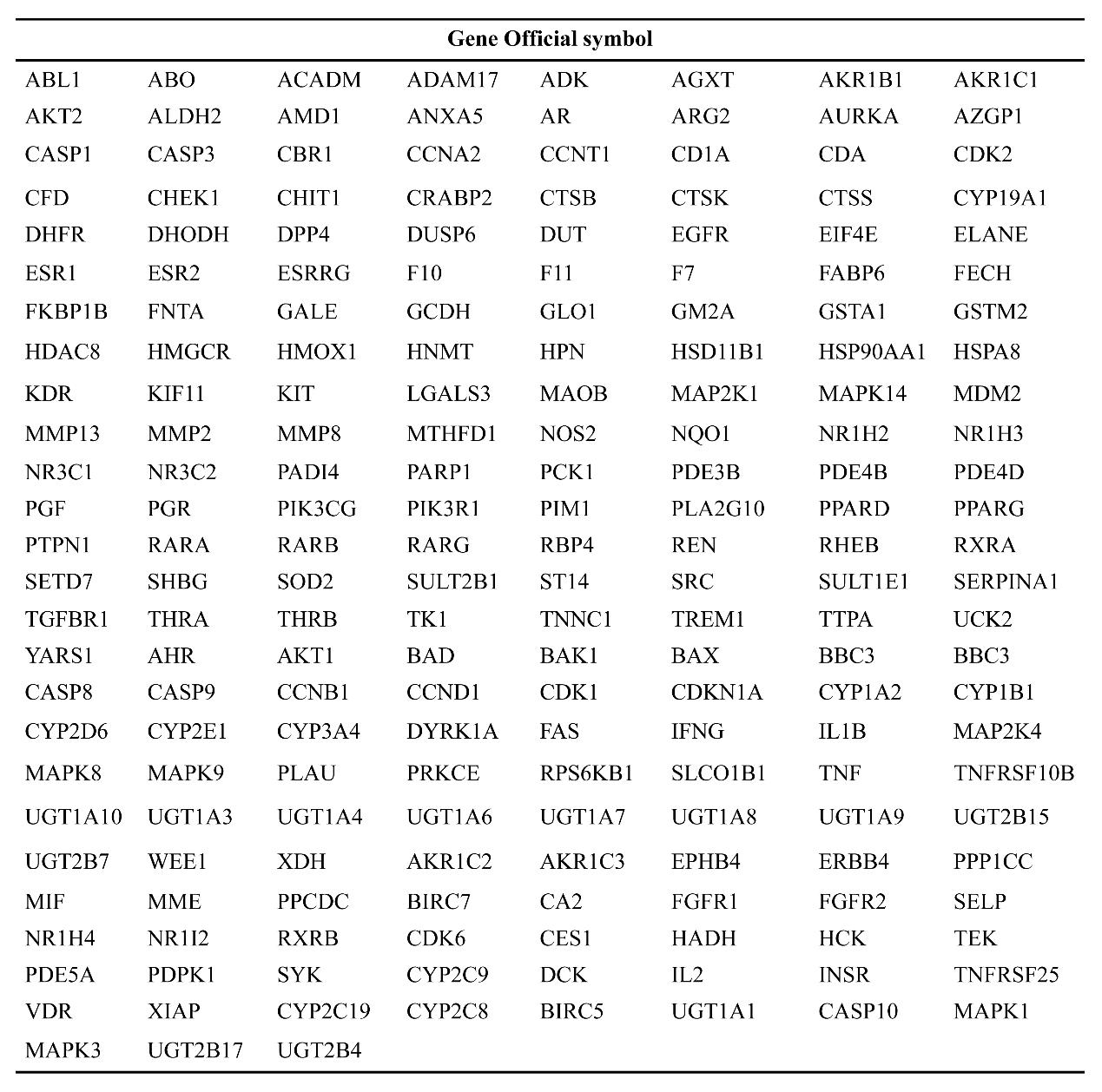


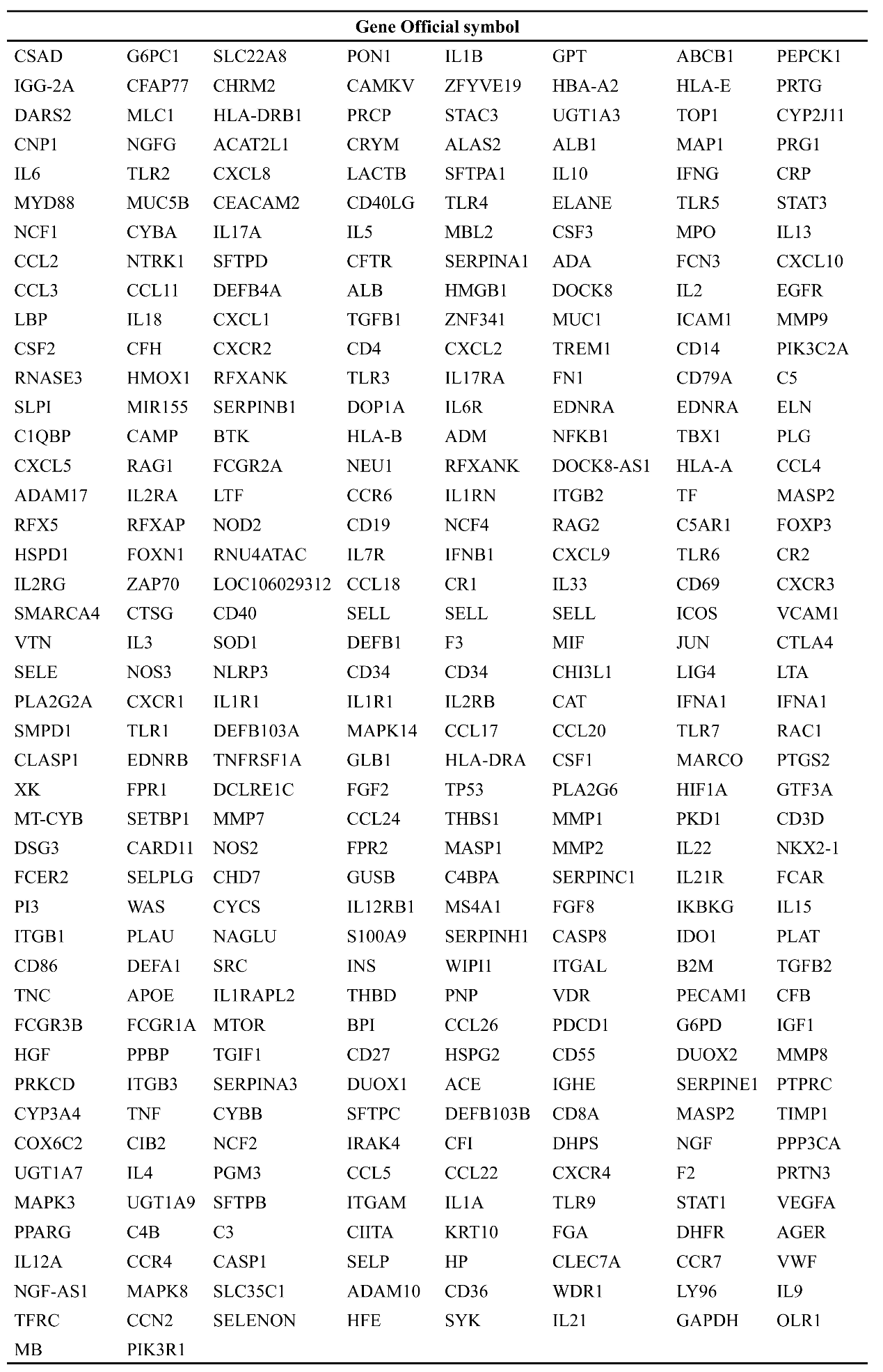
Table 2S. Genes related to *S.aureus* pneumonia.


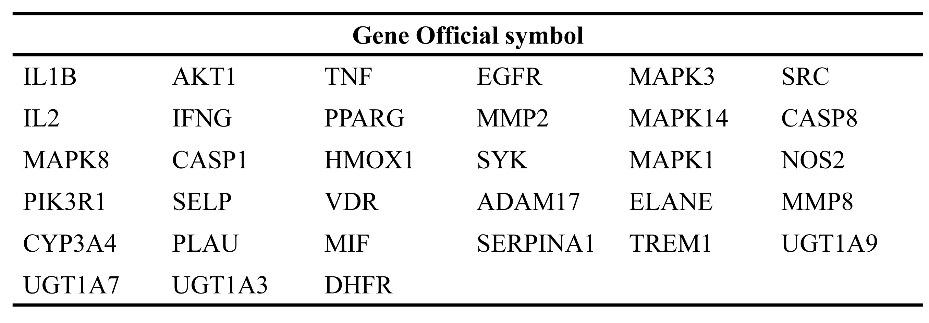
Table 3S Candidate targets of LAA anti-*S.aureus* pneumonia.

Figure 1S. LAA enhanced the phagocyte bactericidal effect against *S. aureus*. The damage induced by PMA-treated human THP-1 cells or with LAA against 29213 was tested after incubation at 37°C for 22 h at different effector to target cell (E:T) ratios. The data are presented as the means ± standard errors (error bars), which were derived from three experiments. The the damage induced by the combination of THP-1 cells and LAA was compared with that in human THP-1 cells alone by the One-Way ANOVA test. ***p* < 0.01 indicates a significant difference between LAA (2 μg/mL, 1 μg/mL) + *S. aureu*s and *S. aureus*/LAA alone.
